# Measurement Matters: A Metrological Approach to Renal Preimplantation Biopsy Evaluation to Address Uncertainty in Organ Selection

**DOI:** 10.1097/TXD.0000000000001708

**Published:** 2024-10-10

**Authors:** John O.O. Ayorinde, Xavier Loizeau, Victoria Bardsley, Spencer Angus Thomas, Marina Romanchikova, Alex Samoshkin, Gavin J. Pettigrew

**Affiliations:** 1 Department of Surgery, University of Cambridge, Addenbrooke’s Hospital, Cambridge, United Kingdom.; 2 National Physical Laboratory, Teddington, United Kingdom.; 3 Department of Histopathology, Addenbrooke’s Hospital, Cambridge, United Kingdom.; 4 Office for Translational Research, School of Clinical Medicine, University of Cambridge, Cambridge, United Kingdom.

## Abstract

**Background.:**

Preimplantation biopsy combines measurements of injury into a composite index to inform organ acceptance. The uncertainty in these measurements remains poorly characterized, raising concerns variability may contribute to inappropriate clinical decisions.

**Methods.:**

We adopted a metrological approach to evaluate biopsy score reliability. Variability was assessed by performing repeat biopsies (n = 293) on discarded allografts (n = 16) using 3 methods (core, punch, and wedge). Uncertainty was quantified using a bootstrapping analysis. Observer effects were controlled by semi-blinded scoring, and the findings were validated by comparison with standard glass evaluation.

**Results.:**

The surgical method strongly determined the size (core biopsy area 9.04 mm^2^, wedge 37.9 mm^2^) and, therefore, yield (glomerular yield *r* = 0.94, arterial *r* = 0.62) of each biopsy. Core biopsies yielded inadequate slides most frequently. Repeat biopsy of the same kidney led to marked variation in biopsy scores. In 10 of 16 cases, scores were contradictory, crossing at least 1 decision boundary (ie, to transplant or to discard). Bootstrapping demonstrated significant uncertainty associated with single-slide assessment; however, scores were similar for paired kidneys from the same donor.

**Conclusions.:**

Our investigation highlights the risks of relying on single-slide assessment to quantify organ injury. Biopsy evaluation is subject to uncertainty, meaning each slide is better conceptualized as providing an estimate of the kidney’s condition rather than a definitive result. Pooling multiple assessments could improve the reliability of biopsy analysis, enhancing confidence. Where histological quantification is necessary, clinicians should seek to develop new protocols using more tissue and consider automated methods to assist pathologists in delivering analysis within clinical time frames.

## Graphical abstract

**Figure:**
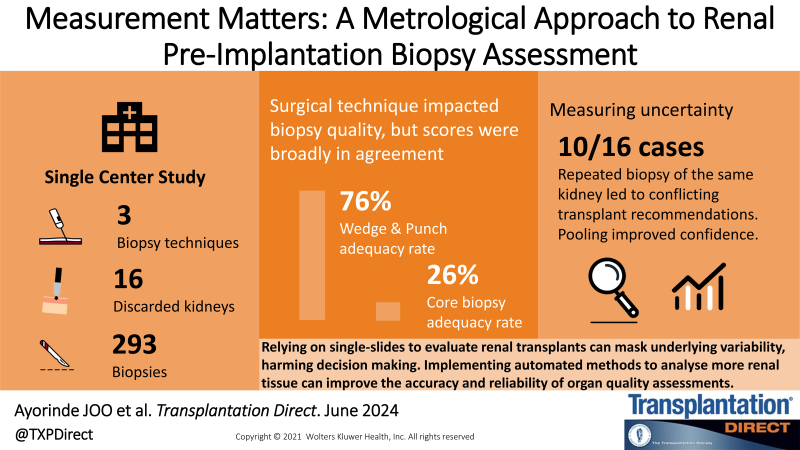

Kidney transplantation is the best treatment for individuals with end-stage renal disease. As the incidence of end-stage renal disease increases,^1-3^ the growing demand for kidneys poses a major challenge.^4^ Over half of potential deceased donors in the United Kingdom are now older than 60 y,^5-8^ and although indiscriminate use of kidneys from older donors may lead to poor transplant outcomes, the discard of potentially suitable organs from this group exacerbates constraints on organ supply.^7-10^

Current methods of assessing kidneys rely on donor history and visual inspection, yet even strongly predictive factors signify cohort rather than individual risk, and there may be significant intracohort variability in outcomes.^6,9,11-15^ Similarly, the relationship between the macroscopic appearance and transplant outcomes remains ill-defined.^16,17^

Quality assessment technologies, such as histopathological analysis or machine perfusion, may aid in organ selection by providing an additional, individualized data point. With histopathological analysis, the decision to implant or discard is informed by assessing the extent of chronic damage present on biopsy.^9,10^ Although validation of this approach through a prospective randomized study is awaited (eg, PITHIA^18^), several studies have reported an association between preimplantation or implantation biopsy assessment and transplant outcome, most notably in Remuzzi’s seminal studies.^19,20^ Consequently, preimplantation biopsy analysis has become widely adopted, with most retrieved organs undergoing biopsy in the United States, up to 78% in some areas.^5,9,21-29^

Remuzzi quantifies each anatomical component of the kidney (glomeruli, tubules, arterioles, and interstitium), providing a total score that determines the implantation strategy (implant each kidney singly into 2 recipients, implant both into 1 recipient, or discard both kidneys). This was devised from a theoretical consideration of providing sufficient nephron mass to the recipient, but its introduction occurred without an in-depth consideration of the metrological aspects of the biopsy procedure, including variability associated with biopsy acquisition and subsequent assessment.^20^ Moreover, to be useful for decision-making, clinicians need to assume that the biopsy score has negligible measurement uncertainty and is representative of the overall quality of the kidney.^30-32^

Awareness of the metrological attributes of a single biopsy result could potentially lead to different implantation decisions. Metrology provides established tools and methods to both quantify and reduce measurement uncertainty,^33-36^ yet these tools have been underused for histopathological assessment. Studies of chronic injury scoring to date have mainly addressed agreement between pathologists,^37-42^ and different surgical approaches to performing the biopsy,^43-45^ but not the variance inherent to scoring, which can only be assessed by repeated measurement of the same subject. Given the widespread use of biopsy analysis and the potential harm from inappropriate use (or discard) of the kidney, this is a surprising oversight with real clinical relevance. In this work, we apply metrological concepts such as comparison and uncertainty to quantify the variabilities in Remuzzi scoring using a series of discarded kidneys.

## MATERIALS AND METHODS

### Biopsy Technique and Slide Preparation

**FIGURE 1.**
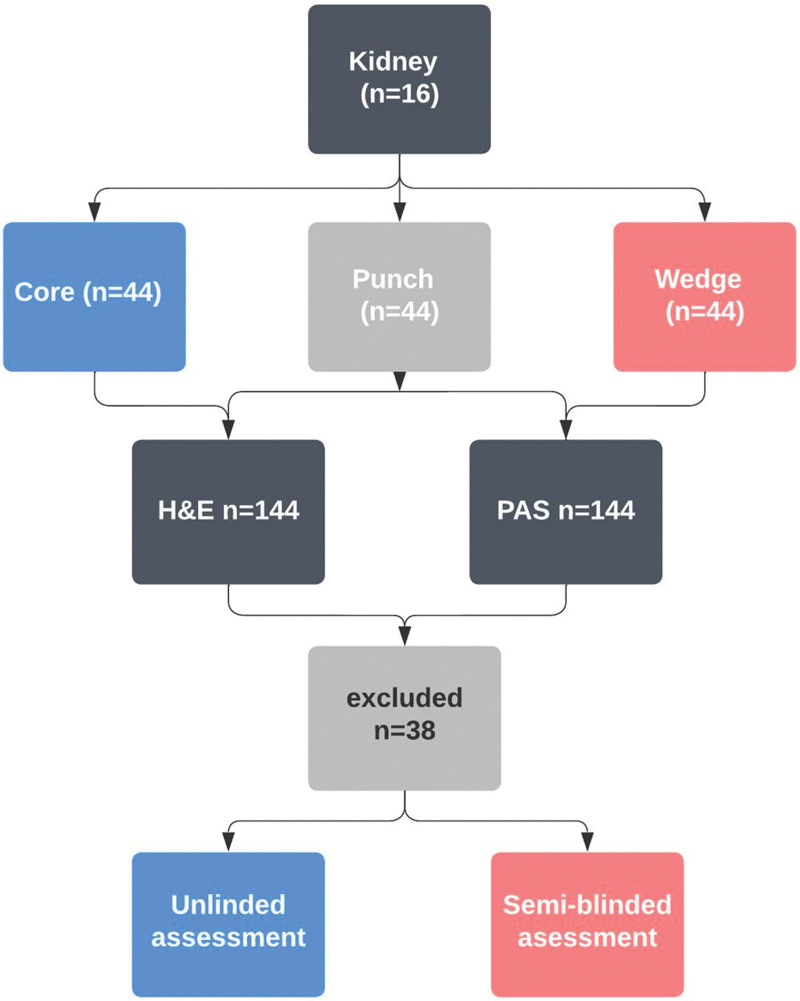
Sample processing and analysis in unblinded and semi-blinded conditions. This flowchart depicts the progression of samples from collection to analysis. For both methods, 12 patients provided 16 kidneys, from which 144 biopsies were produced and 2 HE and PAS glass slides were generated. In the unblinded scenario, slides were examined jointly, and a single Remuzzi score was reported for each biopsy. In the semi-blinded scenario, slides were assessed individually. HE, hematoxylin and eosin; PAS, periodic acid–Schiff.

Two study cohorts were developed: experimental and historical. The experimental cohort involved repeat biopsies on 16 discarded renal allografts (Figure 1). Kidneys were discarded for clinical reasons: donor quality (n = 7), high biopsy score (n = 1), suspected malignancy (n = 3), suboptimal perfusion (n = 2), renal artery thrombosis from retrieval damage (n = 2), and complex cystic lesions (n = 1). Eight surgeons with retrieval experience performed 9 biopsies per kidney (3 core, 3 punch, and 3 wedge), which were then processed into glass slides for digital scanning and assessment.

Core biopsies were performed using a disposable spring-loaded guillotine soft tissue needle (18G 2BioPince Full Core Biopsy Instrument), inserted at a 15° to 20° angle, yielding a 19-mm specimen length. Wedge biopsies involved resecting an ellipse of the renal cortex using a scalpel. Punch biopsies used a 5-mm Schuco Stiefel Biopsy Punch, which limited depth to 8 mm.

Samples were processed according to clinical protocols, that is, placed in 10% neutral buffered formalin and embedded in wax. This mirrors UK practice where biopsies are performed at retrieval by the National Organ Retrieval Service team and processed in parallel with organ transport.^18^

In this study, embedded tissue blocks were sectioned to maximize surface area for slide assessment. Two histological sections from each biopsy were prepared to match time-limited clinical practice: one stained with hematoxylin and eosin (H&E) and the other with Periodic acid–Schiff (PAS). Slides were scanned using a 3DHIS-TECH Panoramic DESK scanner at high resolution (0.12 μm/pixel, minimum 40× objective resolution) and annotated using Sysmex Caseviewer version 2.3.

Out of 288 images from 144 biopsies (Table 1), 38 images were excluded because of inappropriate processing, resulting in folded or incomplete specimens or not displaying the largest cross-sectional area, leaving 250 images for final analysis. To compare the experimental biopsies with those taken for clinical purposes, we developed a comparator cohort of 1044 historical biopsies.

**TABLE 1.** Demographics table for donor (n = 12) and slide (n = 250) characteristics in this study

	Proportion, %	Average (range)
Kidney image library		
DBD	16.7	–
DCD	83.3	–
Cause of death		
Hypoxic brain injury	53	–
Intracranial event	47	–
Sex		
Female	54	–
Comorbidity		
Age, y	–	65.4 (52–76)
Diabetes	8	–
Hypertension	68	–
Smoking	49	–
BMI	–	28.7 (16.5–30.5)
Terminal creatinine, µmol/L	–	73.2 (46–138)

BMI, body mass index; DBD, donation after brainstem death; DCD, donation after circulatory death.

### Biopsy Assessment

We compared unblinded and semi-blinded methods of biopsy scoring. In the unblinded condition, images were assessed on-microscope by an experienced renal histopathologist, as in current practice. This method was considered unblinded because the first assessment might cause anchoring bias on later assessments from the same kidney or patient.^46,47^ To minimize bias, we developed an alternative method of semi-blinded assessment. Digital slides were evaluated by a nonspecialist pathologist, supervised by an experienced renal pathologist at our center. Each evaluation focused on a single component (eg, glomerulosclerosis) on a clean digital slide, with slides evaluated in random order (750 measuring events). Chronic injury features were directly measured on the digital slide, and data were entered into a database to generate the Remuzzi scores concurrently for all slides at the end of the annotation period.

The following measurements were made:

- Glomeruli: all glomeruli present were assessed, determining the proportion of globally sclerotic glomeruli from the total number.
- Arteries: up to 2 arteries per image were assessed, defined as having ≥2 layers of smooth muscle or a diameter >130 μm. If multiple arteries were present, the “worst” artery was used. Arteriosclerosis was determined by the ratio of wall and lumen diameter. Wall thickness was taken as the mean of opposing walls, correcting for nontransverse views. Score boundaries for the lumen-to-wall ratio were converted to definite thresholds to resolve ambiguities in Remuzzi’s original description (0 = 0%–50%; 1 = 50%–80%; 2 = 80%–120%; and 3 = 120%–∞).
- Interstitial fibrosis and tubular atrophy (IFTA): borders were drawn around detected areas of IFTA. We used the approach adopted during the PITHIA trial^18^ of censoring small areas (<5%) of fibrosis to return a score of 0, not 1.
- Adequacy: As in the original description by Remuzzi et al,^20^ adequate images were defined as having at least 25 glomeruli and 1 scorable artery.
- Transplant decision^9^: We used a modification of Remuzzi based on the Cambridge experience; scores 1 to 4 for single transplantation, 5 to 6 for dual transplantation, and ≥7 for discard. This methodology aligns with national protocols and aims to reduce unnecessary discarding and maximize organ utility.^18^

### Statistical Methods

To quantify the strength of the relationship between continuous variables from repeated measurements, we report Pearson’s correlation coefficient (*r*). For numerical measures of agreement for continuous variables, we report the Concordance Correlation Coefficient proposed by Lawrence and Lin,^48^ which accounts for both covariance and deviation from the 45° line of perfect agreement. For categorical variables, we use weighted kappa coefficients.^49^ Strength of agreement is interpreted as follows: 0–0.2 “slight,” 0.2–0.4 “fair,” 0.4–0.6 “moderate,” 0.6–0.8 “substantial,” and 0.8–1 “perfect.”^30^

To compare the sample means of 2 groups, we use the Student *t* test and chi-square test for categorical variables. For comparing sample means for ≥3 groups, we use ANOVA.^50^

To quantify uncertainty and capture the variability in the Remuzzi score, we conducted a bootstrap resampling analysis. This nonparametric approach creates empirical distributions by sampling the experimental population with replacement.^51,52^ Each kidney’s Remuzzi scores were resampled with replacement to create subsamples. We performed 1000 bootstrap iterations, with each subsample containing the same number of scores as the original data. This allowed us to compute statistics such as the mean and SD for each subsample and derive distributions for these without assuming normality.

All tests were considered significant when the *P* value was <0.05. Tests were performed using Python version 3.6 with Numpy version 1.16, Scipy version 1.13, and Scikitlearn version 1.0.2.^53-55^

## RESULTS

### Biopsy Evaluation and Comparison of Surgical Approaches

**FIGURE 2.**
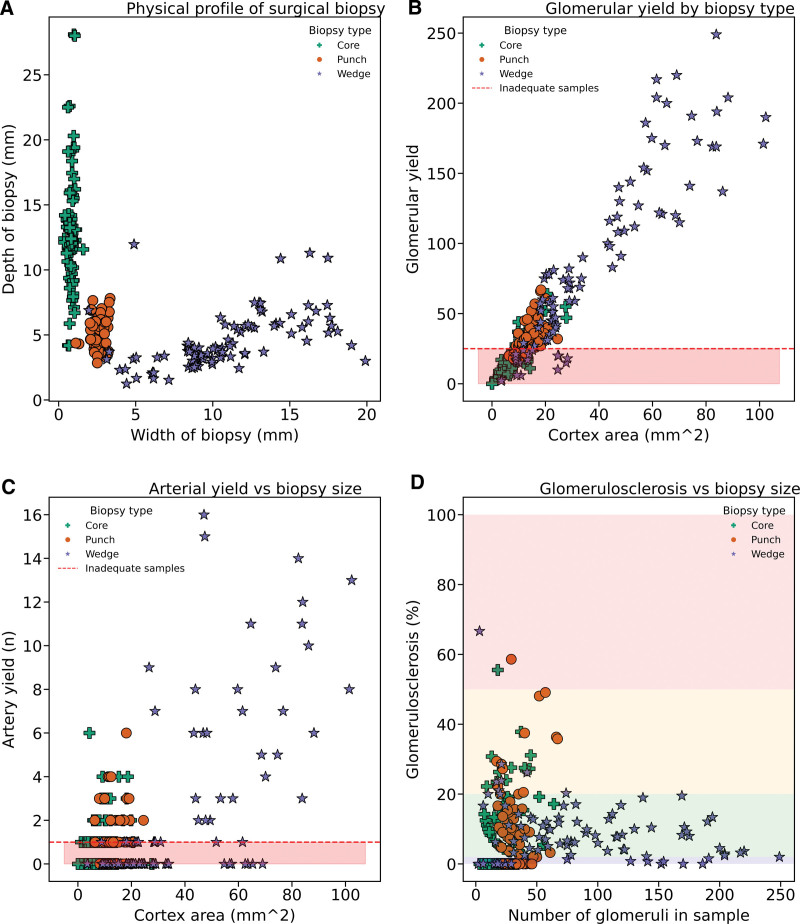
Scatter plots analyzing core (green cross), punch (orange circle), and wedge (purple star) biopsies. A, Physical profile (n = 250), core biopsies are long and thin, with wedge biopsies having the reverse profile. Punch biopsies are the most consistent. B and C, Glomerular yield (n = 250, *r* = 0.94) and arterial yield (n = 249, *r* = 0.62) retrieved and the size of each sample. D, Relationship between the number of glomeruli in the sample and the percentage of glomerulosclerosis. The background color reflects the Remuzzi score (0 = blue, 1 = green, 2 = orange, 3 = red). Glomerulosclerosis is weakly correlated with the size of the sample (Spearman’s rank coefficient *r* = 0.13, *P* = 0.03).

We use measurements from the semi-blinded approach to evaluate the physical characteristics of biopsies obtained through different surgical techniques. The choice of biopsy significantly affected the cross-sectional area available for assessment (Table 2; Figure 2). Core biopsies yielded the smallest samples, whereas wedge biopsies were the largest on average (punch versus wedge, *P* = 0.001; Table 2; Figure 2A). Glomerular yield is correlated with the cross-sectional area (Figure 2B; *r* = 0.94), with core biopsy yielding the fewest and wedge biopsy the most glomeruli. The number of arteries yielded also correlated with biopsy size (Figure 2C; *r* = 0.62), although some large biopsies yielded no arteries. The extent of glomerulosclerosis was only weakly correlated with biopsy size (Figure 2D).

**TABLE 2.** Characteristics of surgical biopsies included in image library (N = 250)

Semi-blind measurements	Core, mean (SD)	Punch, mean (SD)	Wedge, mean (SD)	ANOVA *P*
Width (mm)	**0.8** (0.2)	**2.6** (0.4)	**10.7** (4.03)	**<0.001** ^*a*^
Depth (mm)	**13.0** (4.3)	**5.4** (1.2)	**4.5** (2.1)	**<0.001** ^*a*^
Cortex area	**9.04** (5.1)	**13.7** (3.9)	**37.9** (24.8)	**<0.001** ^*a*^
Vessel count^*b*^	**2.86** (2.5)	**3.5** (2.7)	**7.5** (7.4)	**<0.001** ^*a*^
Glomeruli	**20.2** (12.6)	**32.0** (11.2)	**86.4** (62.9)	**<0.001** ^*a*^
Glomerulosclerosis (%)	**9.4** (11.8)	**11.4** (13.2)	**9.0** (9.2)	0.34
IFTA (%)	**18.1** (22.3)	**21.9** (22.3)	**23.2** (20.8)	0.13
Adequacy rate^*c*^	**26%**	**76%**	**76%**	**<0.001** ^*a*^

^*a*^Following significant ANOVA results, pairwise comparisons showed significant differences in depth, width, total cortical area, and glomerular count between biopsy types. Wedge biopsies were larger, with increased depth, width, and glomerular count. However, no significant difference was found in the mean total cortical area between core and punch biopsies.

^*b*^Vessels include arteries and arterioles.

^*c*^Adequate samples/total samples (%).

IFTA, interstitial fibrosis and tubular atrophy.

Next, we generated Remuzzi scores for each biopsy image, as detailed in the methods section. We grouped and compared the Remuzzi scores for each kidney according to the biopsy technique. This comparison assessed whether the biopsy technique affected Remuzzi scores and, consequently, the transplant recommendation. Weighted Kappa coefficients from paired samples confirmed substantial agreement in assessment across all surgical techniques (Table 3). There was no evidence of a relationship between the surgical method and the resulting transplant recommendation (chi-square test, *P* = 0.41).

**TABLE 3.** Weighted statistics for agreement of Remuzzi scores following each type of biopsy

	Core (weighted κ)	Punch (weighted κ)	Wedge (weighted κ)
Core	–	0.75	0.62
Punch	0.75	**–**	0.78

### Biopsy Quality

Using Remuzzi’s criteria for adequacy,^19,20^ 40% of images in our series were inadequate: 24% failed to meet the glomerular threshold, and 42% had no scoreable artery. Table 2 shows that adequacy varied by biopsy technique, with wedge and punch biopsies achieving adequacy of 76%, whereas only 27.5% of core biopsies were deemed adequate. This is largely due to core biopsies providing insufficient glomerular yield (glomerular adequacy 30%; Figure 2B); arterial adequacy was 52%. There was no discernible pattern of a patient (kidney) or surgeon effect on adequacy rates (**Figure S1, SDC,** http://links.lww.com/TXD/A703).

To ensure that the quality and technique of the biopsies in our experimental cohort were consistent with clinical settings (particularly for wedge biopsies), we compared the physical characteristics of experimental biopsies against a historical cohort of approximately 1000 clinical biopsies taken over a 10-y period (**Figure S2, SDC,** http://links.lww.com/TXD/A703). The physical characteristics and adequacy rates were similar between the experimental and clinical cohorts.

### Assessment Reliability and Quantifying Variability With Bootstrapping

**FIGURE 3.**
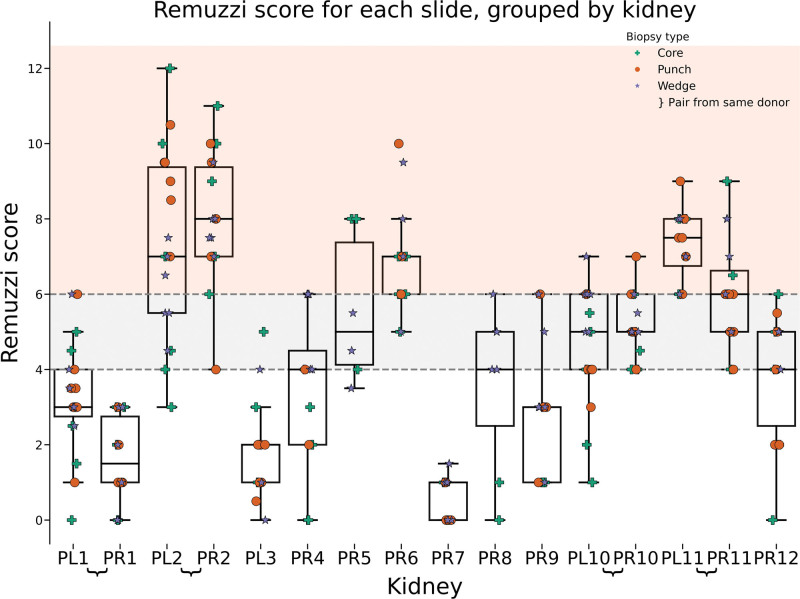
Distribution of Remuzzi scores for each kidney, with each column representing a single kidney (eg, PL1 = donor 1, left kidney). Shading illustrates the decision to transplant singly (1–4, white), as a dual (>4–6, gray), or discard (≥7, red). Markers indicate the type of biopsy performed: green cross for core, orange circle for punch, and purple star for wedge biopsy. Noticeable variation is present within kidney groups, and pairs of kidneys (eg, PL2 and PR2) exhibit similar score distributions.

To maximize the reliability of Remuzzi scoring, we used a semi-blinded approach (Figure 3). Each kidney provided up to 9 samples (stained H&E and PAS), with both kidneys from a single donor being available in 4 instances (donors 1, 2, 10, and 11).

We observed significant variation in Remuzzi scores among repeat samples from the same kidney despite uniform processing and only a single assessor (Figure 3). Notably, for 10 of 16 kidneys, these variations would have led to different clinical decisions regarding implantation or discard. This unexpected and potentially clinically relevant finding emphasized the need to better understand and robustly quantify the observed variability in Remuzzi scores.

In our analysis, we used bootstrapping to quantify variability. Bootstrapping is a powerful statistical technique where random samples are drawn with replacement from the original data. “With replacement” means that an individual data point can be included more than once in each subsample, ultimately generating thousands of simulated data sets. This helps overcome the limited number of repeat samples in our study, allowing for the estimation of population statistics without assuming normal data distribution.

Through bootstrapping, we determined that the variability in Remuzzi scores likely reflects inherent sampling variability as opposed to underlying differences in chronic injury. This was substantiated by statistically similar distributions of scores between left and right kidneys from the same donor (eg, Mann-Whitney test PL10 versus PR10; *P* = 0.37) and similar means and variances in bootstrapped distributions. The range of agreement on transplant outcome within pairs for bootstrapped samples was between 62% and 81% (Table 4, left section). However, statistical dissimilarity was observed when comparing kidneys from different donors (eg, PL/R10 versus PL/R11; *P* = 0.004).

**TABLE 4.**
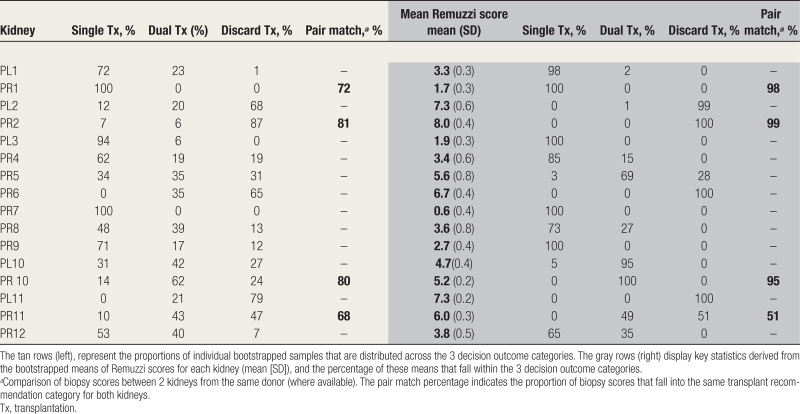
This table presents the result of 2 bootstrapping approaches applied to the data set

**FIGURE 4.**
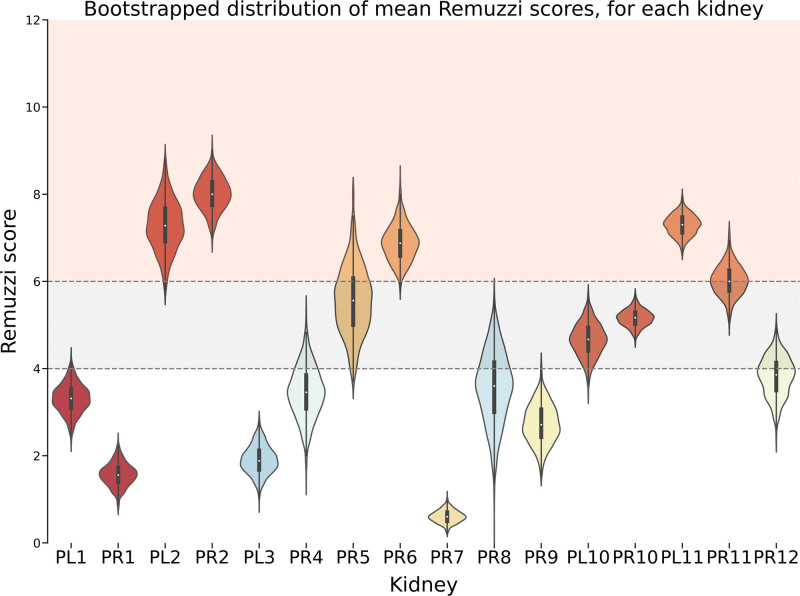
Violin plot representing the bootstrapped (n = 1000) distribution of Remuzzi score sample means for each kidney. The plot presents the spread and density estimation of the simulated means. Shading illustrates the decision to transplant singly (1–4, white), as a dual (>4–6, gray), or discard (≥7, red). Bootstrapped means distributions show a tighter range as well as high similarity distribution within pairs, whereas distributions from other donors are generally less alike.

Furthermore, bootstrapping demonstrated that incorporating multiple measurements can reduce measurement uncertainty and enhance confidence. Each kidney exhibited a unique probability distribution of Remuzzi scores for each sampled set, consistent between surgical methods (**Figures S3–S6, SDC,** http://links.lww.com/TXD/A703). For some kidneys (eg, PR7), this distribution was narrow, whereas for others (eg, PR5), sample scores were more widely spread across decision thresholds. We propose that this may represent differing levels of “confidence” in determining the most appropriate outcome. Using the mean of each sampled set as the estimate (Figure 4) reduced the spread of estimates and the proportion of assessments crossing decision-making thresholds (Table 4, left section versus right), leading to increased agreement on clinical recommendations. The exception was pair P11, which demonstrates the rule: assessment means and variances for each kidney were similar, but because the distribution of PR11 lies in close proximity to the upper Remuzzi threshold, there were a substantial proportion of scores crossing categories, causing within pair disagreement (Table 4, right section, pair match 51%).

### Pathologist Validation of Reliability Findings

The semi-blinded assessment was implemented to mitigate anchoring effects and to serve as a bridge to automated slide analysis.^56^ However, this approach raised concerns about the introduction of new biases, particularly in the assessment of IFTA—where normally visual approximation is used instead of measurement of bounded pixel area—and arteriosclerosis, where the boundaries between classes in Remuzzi’s framework are ambiguous.

**FIGURE 5.**
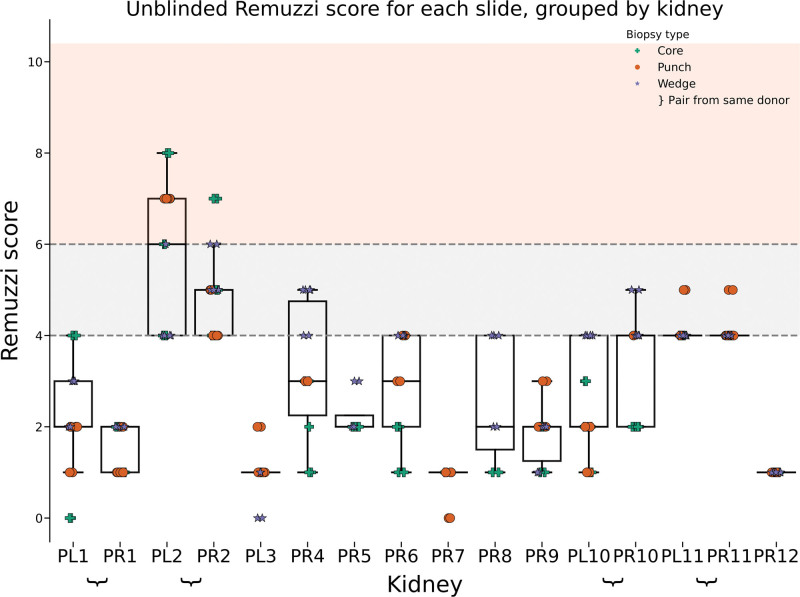
Distribution of unblinded (glass slide) Remuzzi scores for each kidney, with each column representing a single kidney (eg, PL1 = patient 1, left kidney). Shading describes the decision to transplant singly (1–4, white), as a dual (>4–6, gray), or discard (≥7, red).

To evaluate the validity of semi-blinded assessments, a renal histopathologist independently assessed the original glass slides (Figure 5). Notably, the pathologist’s assessments were conducted using both PAS and H&E stains, which is in line with clinical practice, effectively halving the number of assessments compared with the semi-blinded approach. Despite every advantage, given the lack of blinding and making fewer assessments, we found significant assessment variability for individual kidneys, which crossed decision boundaries in 6 out of 16 cases. Because increasing the number of assessments increases the chance of any 2 assessments disagreeing, the reduced number of instances where decision boundaries were crossed might simply be attributable to the fact that the pathologist assessed fewer cases. Importantly, we observed a similar scoring pattern for each kidney, with similar median scores to those of the semi-blinded cohort.

### Visualization and Analysis of Consistency Between Semi-blinded and Unblinded Methods

**FIGURE 6.**
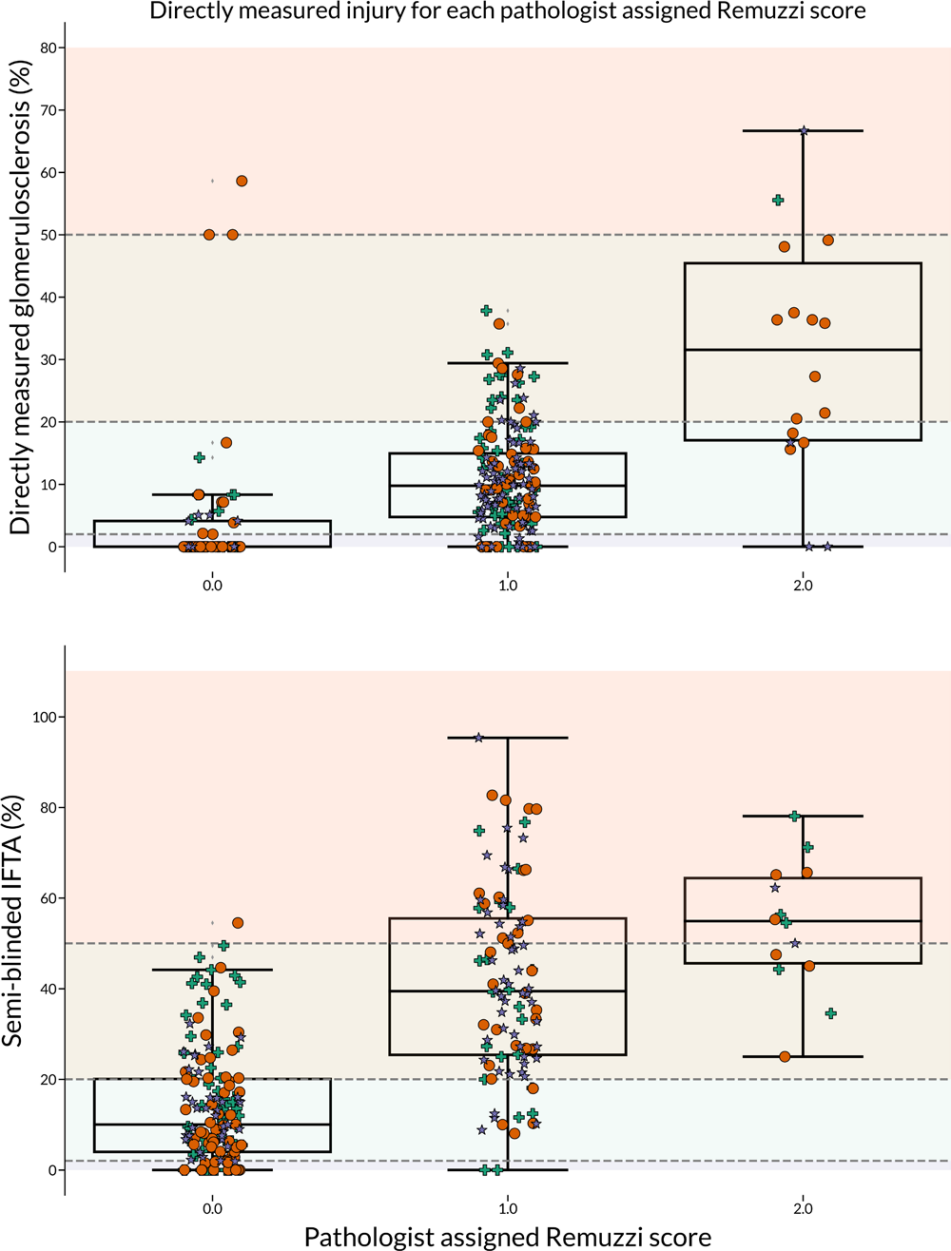
Box plots that show the measured degree of injury on the y-axis for each slide scored by the pathologist (n = 265). Glomerulosclerosis = upper, IFTA = lower. The perfect agreement would show increasing, nonoverlapping groups, which are separable at the Remuzzi threshold for each component (gray boundaries 0–3). IFTA, interstitial fibrosis and tubular atrophy.

To examine the consistency between the unblinded and semi-blinded assessment methods, we categorized each digital slide based on the injury category assigned by the pathologist (0–3) and visualized the measurements made for that slide (Figure 6). For this analysis, we focused on glomerulosclerosis and IFTA, as these are continuous measurements that apply to the entire slide rather than a specific object, such as arteriosclerosis. The Remuzzi score for IFTA comprises 2 separate assessments—interstitial fibrosis and tubular atrophy. Although we derived separate values for interstitial fibrosis and tubular atrophy, these scores did not diverge between the experimental and clinical cohorts. Hence, they are considered as a single measure of injury.

In the case of perfect agreement between the 2 assessment methods, one would have expected data points to cluster within the Remuzzi classes of injury, delineating each decision boundary. However, for both glomerulosclerosis and IFTA, we observed that although average injury measurements did increase with the assigned category of injury, there was significant overlap between classes.

## DISCUSSION

### Impact of Surgical Technique on Biopsy Quality and Assessment

Surgical technique may influence biopsy assessment because of differences between the physical profiles and sampling locations of each method.^25,31,38-41,43,44,57-59^ Core biopsy, the standard approach, has 2 main disadvantages: it provides small volumes of tissue and skill is required to avoid damage to hilar structures or sampling medulla rather than cortex. The main alternative is wedge biopsy, where a scalpel is used to resect an ellipse of cortex. However, without standard measurements or anatomical landmarks, samples vary greatly. A minority are “safe,” but small, subcortical resections that are not useful for quality assessment, whereas extremely large, deep biopsies risk the safety of the transplant. In 2012, Bago-Horvath et al^45^ proposed the punch biopsy, which provides a consistent barrel of renal cortex and limits insertion safe depth to 8 mm. A punch biopsy is not widely used despite these advantages, and current knowledge is limited to retrospective comparisons with historical cohorts. Differences in sampling location may also introduce bias. For instance, Muruve et al^43^ found that small wedge biopsies overestimate glomerulosclerosis by sampling the subcapsular region where sclerosed glomeruli are more prevalent, a finding confirmed by Rule et al.^60^ Researchers are yet to reach a consensus on the best surgical method. Core and wedge biopsies have been extensively studied, with some groups advocating core biopsies for evaluating vascular lesions,^40,57,61^ whereas others have favored wedge biopsies for predicting clinical outcomes.^44,62^

To our knowledge, this is the first prospective comparison of all 3 biopsy methods. Despite theoretical concerns, our analysis found substantial agreement between each method (Table 3), aligning with a comparison done by Mazzucco et al^41^ of “core” and “wedge” biopsies to whole kidney sectioning (κ = 0.57 wedge versus section; κ = 0.73 core versus section score). However, the choice of technique did affect the quality of the sample provided. Core biopsies consistently produced the smallest samples (Figure 2A), and in our study, that led to a slide adequacy of only 26% because of the correlation between biopsy size and glomerular yield (Figure 2B). Inadequate sampling is a concern because of the risk of a repeat biopsy causing unnecessary complications and prolonging cold ischemia.

### Reproducibility

Our study revealed significant variation in chronic injury on repeated assessment, which persisted whether the slide was assessed as standard or by direct measurement of chronic injury features. The magnitude of variability was clinically significant and would have led to contradictory recommendations regarding utilization or discard. Signs of this phenomenon have been recognized previously (eg, left versus right discrepancy), but the cause has not been reliably identified.^63^ Numerous studies have remarked on inconsistent agreement between pathologists.^30^ Indeed, pathologist experience has been repeatedly identified as a factor influencing the degree of interobserver agreement, suggesting that variability can be reduced by training or experience.^37,39^

In contrast, the role of measurement in variability has been underexplored. When scoring, discretion is used in selecting and measuring, which can introduce variation. In the previous example, measurement style might vary between specialist renal and general pathologists. Additional variation could arise from heterogeneity of disease expression in the kidney cortex, fixation effects, staining impacts on injury visibility, time constraints, or subjective understanding of assessment criteria.

Our analysis suggests that each slide contributes an estimate of chronic injury within a probability distribution. The quality of the kidney is, therefore, represented by the distribution of scores it receives rather than by any single result. This better reflects reality and, usefully, provides a way of quantifying assessment confidence. We propose that confidence is inversely related to the percentage of estimates that cross decision boundaries, such that high confidence levels are seen with a narrow distribution of scores within a single injury category. Using this framework, we could demonstrate the value of combining multiple measurements (pooling) to reduce variance (Table 4), thus increasing confidence and reducing the frequency of contradictory recommendations within kidney pairs. Confidence could be used in future studies to guide interventions aimed at minimizing variability. Additionally, our approach addresses variability in clinical settings because our data support clinicians in avoiding overly dogmatic interpretations of chronic injury assessment and attributing differences in Remuzzi scores (particularly within pairs) solely to biological factors.

### Limitations

Discarded kidneys were necessary to allow repeat biopsies, but their use may limit the generalizability of our findings. Although we assessed a comparable number of biopsy slides to other major studies in this area (n = 250), they were derived from only 16 kidneys, potentially introducing bias. Future studies should systematically assess hundreds or thousands of slides using supervised automated assessment methods.^56,64,65^ Moreover, the 18-gauge needles used in this study reflected National Organ Retrieval Service practice per the QUOD protocol and may have reduced sample quality compared with 16-gauge needles, which provide better tissue sampling.^66-69^ Future work should use 16-gauge needles to improve sample quality.

As the kidneys were not transplanted, we could not assess the impact of large wedge biopsies on transplantability or bleeding, which are important clinical concerns. Additionally, our methods may not fully align with clinical practices. For example, in hours, our center processes and stains biopsy at a minimum of 10 levels, allowing pathologists to search for an artery within the stack if one is not found on the initial slide. By reporting each slide individually, we may overestimate the clinical likelihood of an inadequate sample for the whole biopsy, potentially explaining the disparity in adequacy rates between our clinical and experimental cohorts (approximately 95% versus 75%; **Table S1, SDC,** http://links.lww.com/TXD/A703).

Finally, our analysis relied solely on the Remuzzi assessment score. While other scoring systems, such as Banff, are used in transplant pathology, we selected Remuzzi for its focus on chronic injury features (versus rejection) and organ evaluation. Remuzzi offers clear thresholds, simplicity, and potential compatibility with other donor risk assessments. Nonetheless, our metrological insights should be applied to any histopathological quantification method.

### Implications for Future Practice

Unlocking the full potential of biopsy assessment requires a deeper understanding of measurement error. Systematic investigations into biopsy metrology are needed to identify and reduce the major contributors to variation. Remuzzi’s original description contains obvious candidates for study and optimization, including

- that the analyzed tissue safely represents the entire organ,
- the chosen measurands appropriately predict organ quality,
- the thresholds dividing scores effectively separate organs with differing quality,
- the measured quantities are combined and weighted optimally,
- measurement uncertainties do not affect decision-making.

The impact of metrology on interpretation is clear. If biopsy scores are highly reproducible, then kidneys with conflicting scores from the same donor should have different implantation strategies. However, given the observed low reproducibility as in this study, differences could merely reflect variance. Clinicians should use both sources of information and average contradictory scores unless there are clinical indicators to suspect unilateral dysfunction (eg, large size discrepancy).

Pooling samples is a promising strategy to address the deficiencies of single-slide assessment, yet to achieve high levels of reliability (ie, >95% chance repeat testing gives an equivalent score, or >99% chance the same decision) means analyzing much more tissue than currently. However, implementing multi-slide and multi-biopsy protocols using traditional methods may not be practical. Advancements in digital pathology and automation offer an opportunity to reconsider the time and effort available for urgent biopsy analysis.^56,65,70-77^ Interdisciplinary collaboration among clinicians, metrologists, and data scientists could facilitate the introduction of technologies to greatly improve decision-making. Automated slide analysis could transform the gold standard of pathology review, reduce variability, and improve predictive power through exhaustive whole-biopsy analysis,^64^ working within time constraints by performing assessments in parallel.^56,65,70^

## CONCLUSIONS

Our study highlights the limitations of relying on single-slide assessments for organ selection. We observed that biopsy quality is significantly influenced by surgical technique and demonstrated the need to identify and reduce other sources of variation. While acknowledging the limitations of this study, our findings suggest that there are opportunities to improve histological assessment as a quantification tool through a comprehensive evaluation of histopathological practices, optimization of current assessment systems, and rigorous metrological analysis. Advances in computer vision could enable the analysis of much more renal tissue within clinical time frames.

Improving measurement standards to the level seen in other areas of routine medical care (eg, blood tests) will likely be cost-effective and greatly improve patient outcomes.

## ACKNOWLEDGMENTS

This work would not have taken place without the determined support of the Office for Translational Research, University of Cambridge.

## Supplementary Material


