# Supplementary material for: Measurement Matters: A Metrological Approach to Renal Preimplantation Biopsy Evaluation to Address Uncertainty in Organ Selection
Source: Transplant Direct. 2024 Oct 10;10(11):e1708. doi: 10.1097/TXD.0000000000001708 (PMC11469905; doi:10.1097/TXD.0000000000001708)
Supplement: Supplementary file 1 [file txd-10-e1708-s001.pdf]

Supplementary

1 Table of Contents

2 Surgeon effect 2

3 Clinical validation 3

4 Bootstrapped Remuzzi scores 5

Figure S3-6: Distribution of raw bootstrapped values in the experimental cohort

2 Surgeon effect

There was no consistent pattern across biopsies in the sizes of the samples taken by different surgeons. For wedge biopsies, which were the most variable, surgeons procured samples with significant overlap of size distributions (Surg5-8), some surgeons retrieved mostly small (Surg2&3) or large (Surg4) biopsies, and others still were highly variable in their technique (Surg1). However, this may reflect the differing number of samples performed by each surgeon. The effect of the punch biopsy technique on variability can be seen in how it collapses variability into one dimension

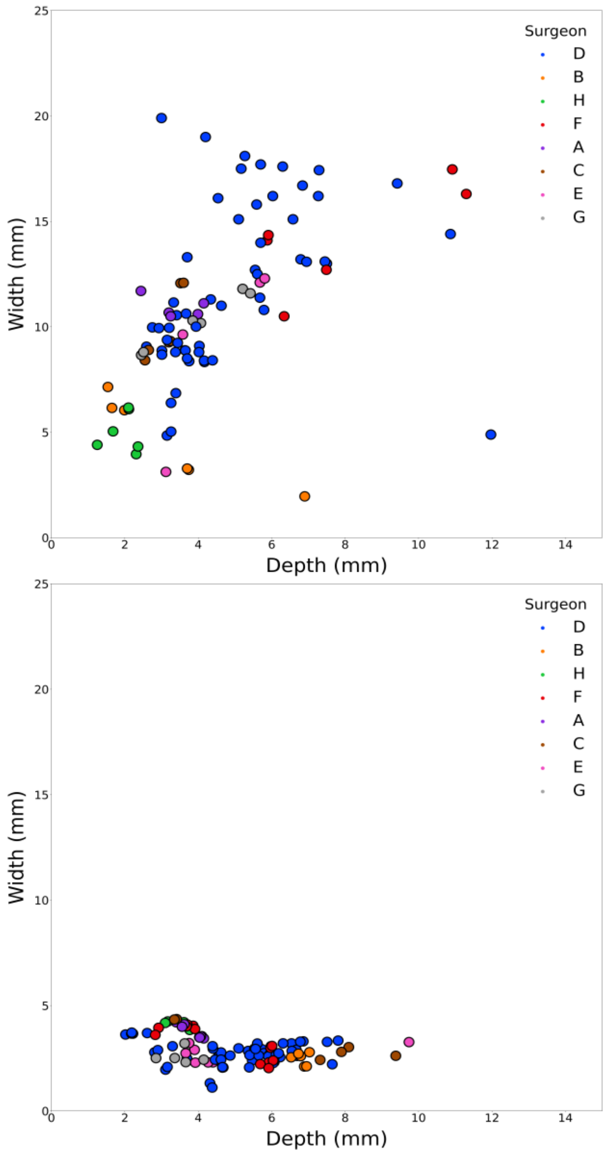

Figure S1: Scatter plot of dimensions of the biopsies returned by each surgeon (colour). (upper) wedge biopsies, very small (inadequate) & very large (dangerous) biopsies possible. (lower) punch biopsy variability largely confined to depth of the sample, returning more consistent biopsy size.

3 Clinical validation

An important concern in extrapolating from assessments made on discarded kidneys to the clinical environment was that less care might have been taken by surgeons in performing biopsies. We the therefore compared the physical characteristics of the experimental cohort with historical biopsies performed at our institution. As part of usual care, the Cambridge transplant service has an ongoing pre-implantation biopsy service since 2009. The original reports for these biopsies were available. The clinical cohort contained 1044 biopsies, from donors with an average age of 56.8 years (sd 16.2). Most of these samples were wedge biopsies (n=970), whilst punch (n=29) and core (n=22) biopsies made up a minority of samples; a small number of biopsies did not have reports (n=23).

The average size of wedge biopsies were similar between the clinical and experimental cohorts, although punch (clinical = 25.5 mm^2 sd 15.1 vs experimental = 13.7 mm^2 sd 3.9) and core (clinical = 17.6 mm^2 sd 9.1 vs experimental = 9.04 mm^2 sd 5.1) biopsies were slightly larger. Relationships between glomerular yield, glomerulosclerosis, and biopsy size were as in the experimental cohort. Wedge (94.3%) and Punch (96.6%) biopsies yielded a greater proportion of adequate samples in the clinical cohort compared to core (63.6 %).

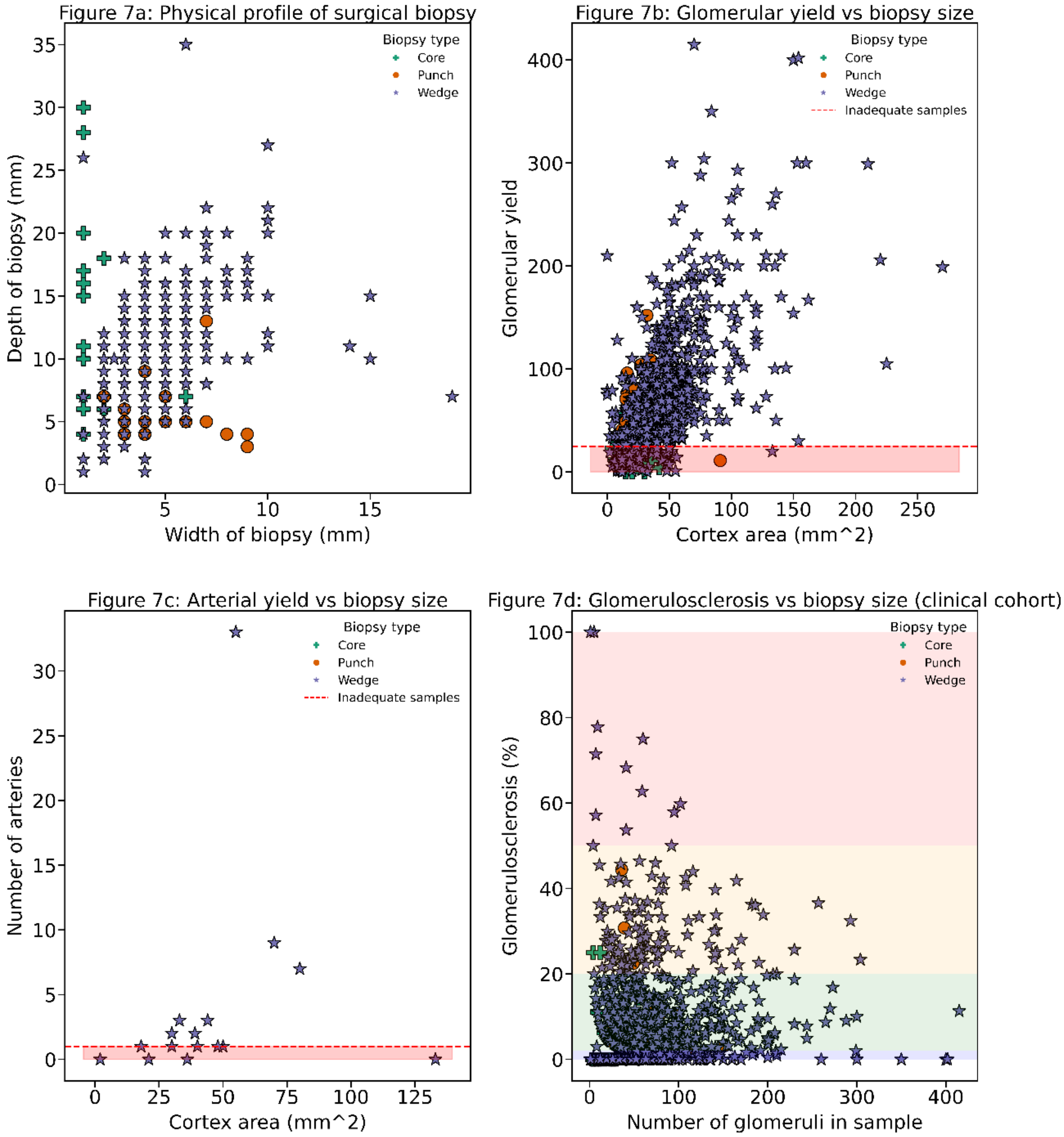

Figure S2: Scatter plots analysing core (green cross), punch (orange circle) and wedge (purple star) biopsies in the clinical cohort. 7a) Physical profile (n=1044), similar physical profile allowing for inconsistencies in labelling dimensions. 7b) & 7c) Glomerular yield & Arterial yield retrieved and the size of each sample, few reports contained specific count for arteries. 7d) Glomerulosclerosis pattern is similar as for the experimental cohort. Background shading demonstrates each Remuzzi score assigned.

4 Bootstrapped Remuzzi scores

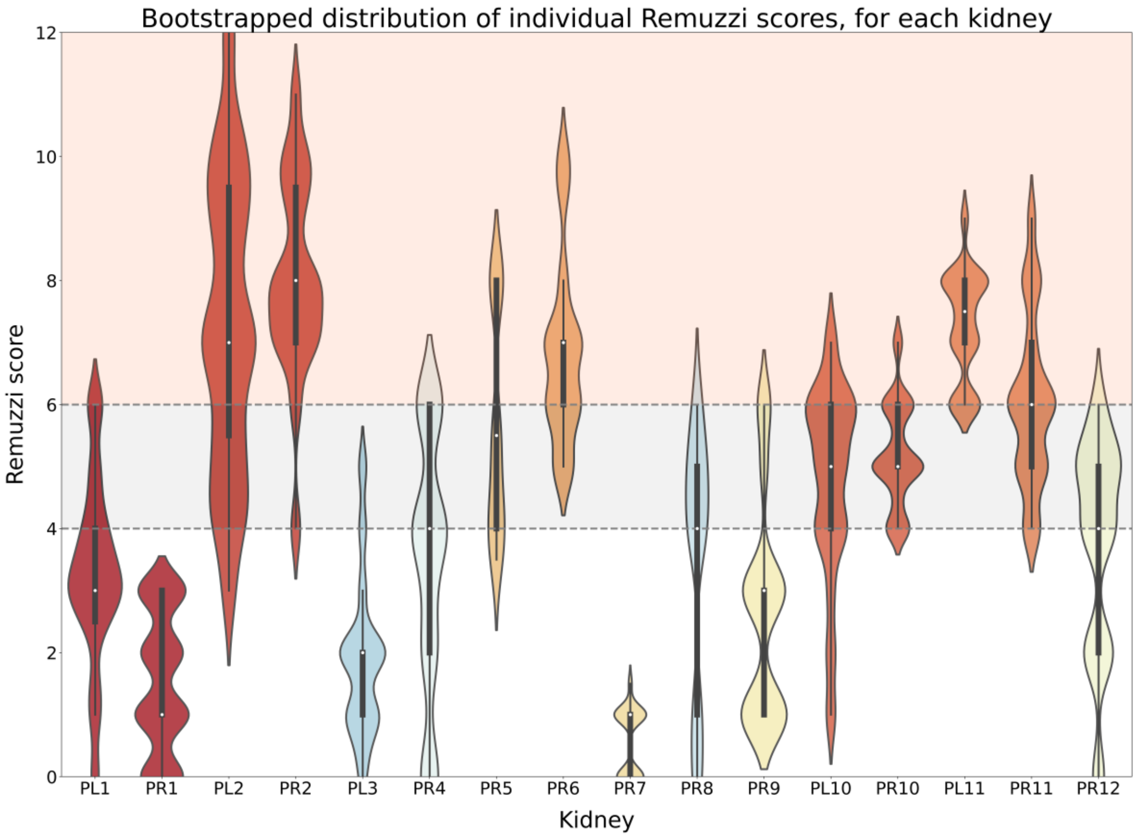

Figure S3: Violin plot representing the raw bootstrapped (n=1000) Remuzzi scores sample means for each kidney. The plot presents the spread and density estimation of the simulated means. Shaded areas denote the transplant strategy (white=single, grey=dual, red=discard). Bootstrapped means distributions show a tighter range as well as high similarity distribution within pairs, whilst distributions from other donors are generally less alike.

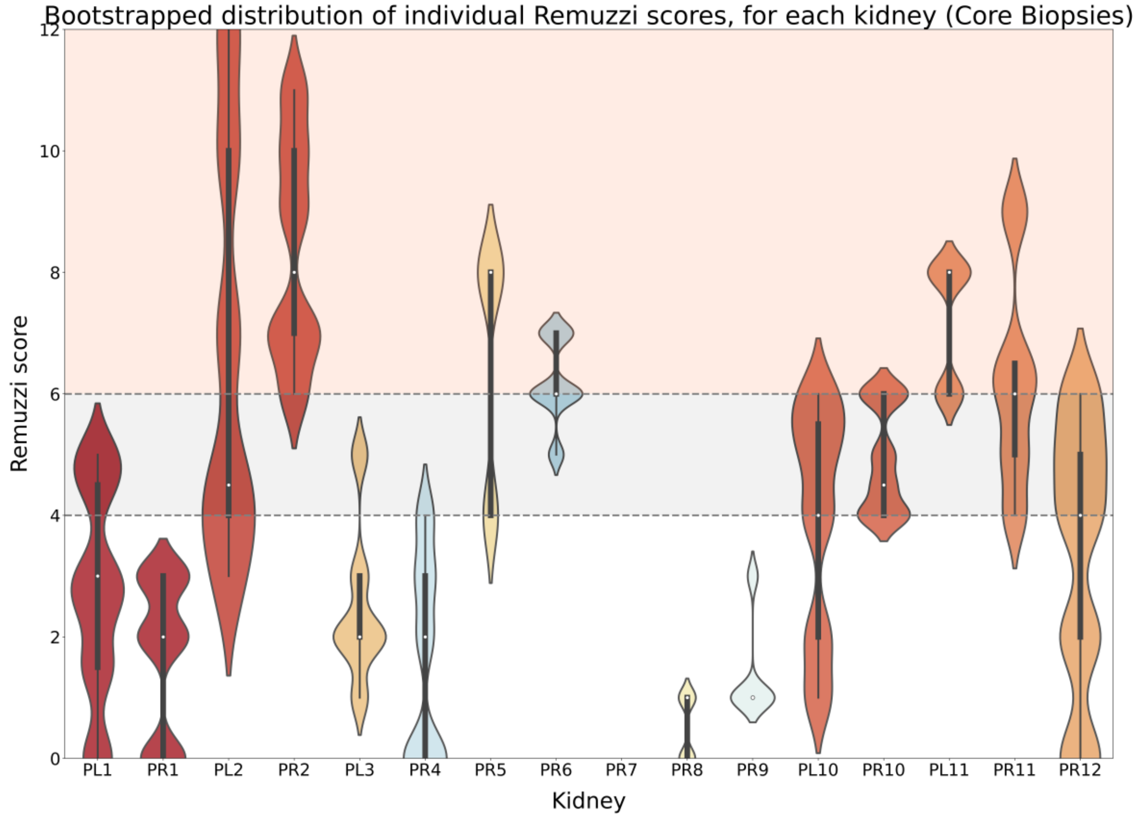

Figure S4: Violin plot representing the raw bootstrapped (n=1000) Remuzzi scores sample means for core biopsies of each kidney. The plot presents the spread and density estimation of the simulated means. Shaded areas denote the transplant strategy (white=single, grey=dual, red=discard). Bootstrapped means distributions show a tighter range as well as high similarity distribution within pairs, whilst distributions from other donors are generally less alike

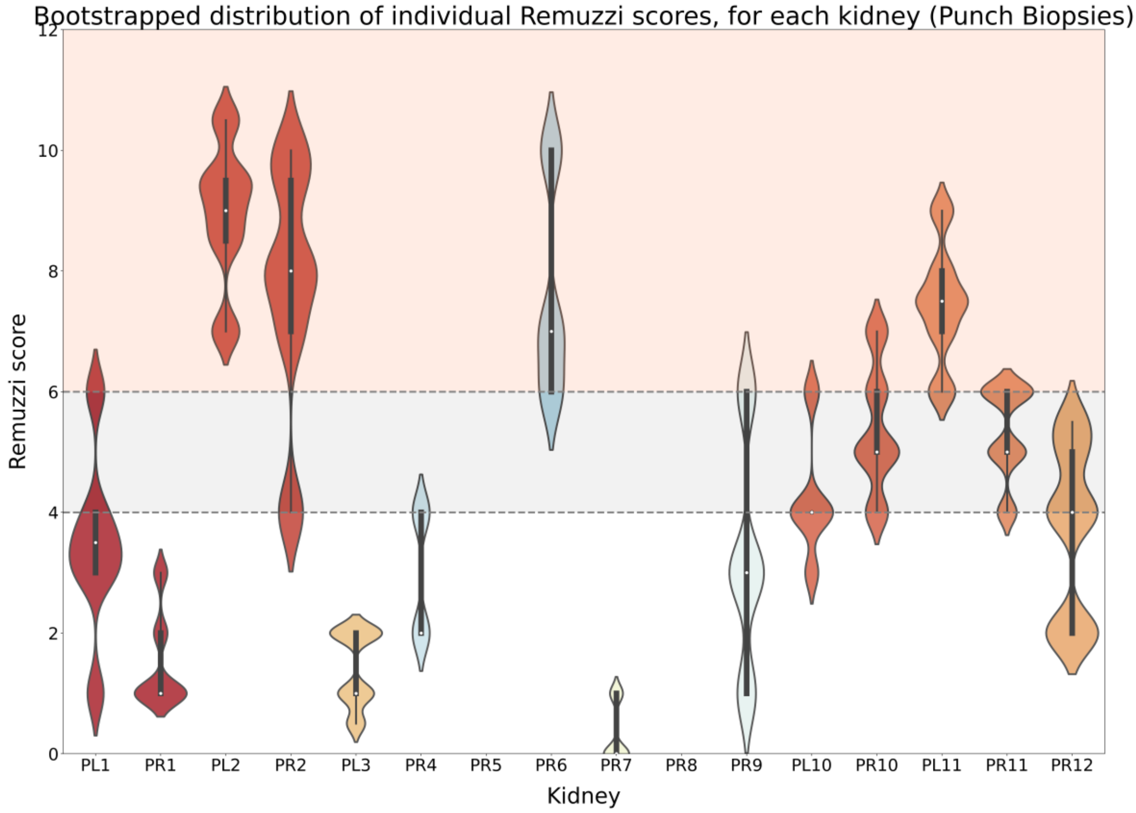

Figure S5: Violin plot representing the raw bootstrapped (n=1000) Remuzzi scores sample means for punch biopsies of each kidney. The plot presents the spread and density estimation of the simulated means. Shaded areas denote the transplant strategy (white=single, grey=dual, red=discard). Bootstrapped means distributions show a tighter range as well as high similarity distribution within pairs, whilst distributions from other donors are generally less alike

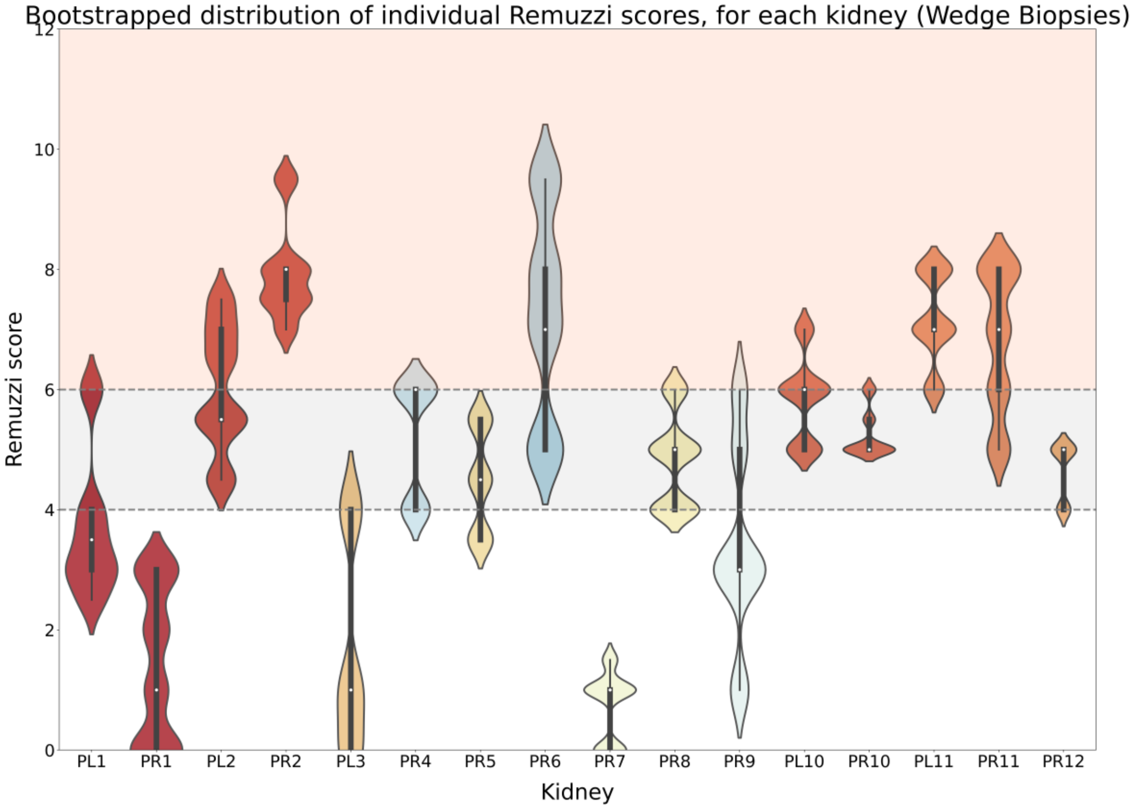

Figure S6: Violin plot representing the raw bootstrapped (n=1000) Remuzzi scores sample means for wedge biopsies of each kidney. The plot presents the spread and density estimation of the simulated means. Shaded areas denote the transplant strategy (white=single, grey=dual, red=discard). Bootstrapped means distributions show a tighter range as well as high similarity distribution within pairs, whilst distributions from other donors are generally less alike

Table S1. Characteristics of surgical biopsies in clinical cohort (n=1044)

	Core (mean;sd)	Punch (mean;sd)	Wedge (mean;sd)	ANOVA p value
**Kidney image library**				
Width (mm)	**1.4** (1.1)	**4.7** (1.9)	**9.8** (3.6)	**<0.001***
Depth (mm)	**14.6** (9.0)	**5.3** (1.9)	**4.29** (1.68)	**<0.001***
Cortex area (mm^2)	**17.6** (9.3)	**25.2** (15.7)	**44.9** (30.1)	**<0.001***
Vessel count	--	--	**3.3** (7.4)[1]	NA
Glomeruli	**12.8** (13.6)	**61.0** (33.6)	**69.9** (56.8)	**<0.001***
Glomerulosclerosis (%)	**5.9** (9.73)	**11.8** (25.2)	**9.6** (12.0)	**0.04***
IFTA (%)[1]	--	--	--	NA
Remuzzi score	**1.6**	**2.3**	**1.9**	0.06
Adequacy rate[2]	**64%**	**97%**	**94%**	**--**

[1] Given on a minority (n=20) of samples, [2]Adequate samples/Total samples (%)

*Pair comparisons following significant ANOVA tests showed that Depth and width varied significantly between all pairs, except Punch-Wedge in depth (p > 0.05). Total cortex area and glomerular count differed significantly in Core-Wedge and Punch-Wedge comparisons but not in Core-Punch. Percentage of sclerosis did not significantly differ between any individual pairs despite the significant ANOVA test.
